# Optimizing harvest stage and drying time to enhance yield and nutritive quality of whole-plant *Tithonia diversifolia* forage meal in arid tropics

**DOI:** 10.3389/fpls.2025.1644949

**Published:** 2025-09-19

**Authors:** Victor Temoche-Socola, Emmanuel Sessarego, Aníbal Rodríguez, Cesar Vásquez, Joel Riojas, José Ruiz, Juancarlos Cruz

**Affiliations:** ^1^ Estación Experimental Los Cedros, Instituto Nacional de Innovación Agraria (INIA), Tumbes, Peru; ^2^ Universidad Nacional de Tumbes, Departamento Académico de Medicina Veterinaria y Zootecnia, Facultad de Ciencias Agrarias, Tumbes, Peru; ^3^ Dirección de Supervisión y Monitoreo en las Estaciones Experimentales Agrarias, Instituto Nacional de Innovación Agraria (INIA), Lima, Peru

**Keywords:** nutritive value, digestibility, forage potential, phenological stage, leaf-to stem ratio

## Abstract

**Introduction:**

*Tithonia diversifolia*, also known as Mexican sunflower, is a tropical shrub with high forage potential, but limited information exists on the optimal harvest stage to balance yield and nutritional value.

**Methods:**

A 3 × 3 factorial design was implemented under tropical dry forest conditions in northern Peru, combining three regrowth stages (30, 45, and 60 days) with three plant fractions (leaf, stem, and leaf–stem mixture), with four replicates per treatment. Agronomic traits, dry matter yield, proximate composition, and in vitro dry matter digestibility (IVDMD) were evaluated.

**Results:**

Biomass yield increased significantly with harvest age, reaching 11.93 kg fresh weight and 3.45 kg dry matter per plant at 60 days, although with reduced nutritional quality due to a higher stem proportion. Leaves harvested at 30 days had the highest crude protein (16.5%) and soluble carbohydrates (48.2%) with the lowest crude fiber (19.3%). In contrast, the 45-day leaf samples achieved the highest IVDMD (62.34 ± 1.42%). A strong positive correlation was observed between leaf area and biomass yield (r = 0.93), and a moderate negative correlation with digestibility (r = –0.42).

**Discussion:**

Harvesting at 45 days provided the best compromise between biomass production and nutritional value. These findings highlight the potential of *T. diversifolia* as a strategic forage alternative for sustainable feeding systems in tropical dry environments.

## 1 Introduction

Forage scarcity in tropical dry forest regions represents one of the main constraints on livestock production, especially in extensive goat systems. This situation, exacerbated by climate variability and soil degradation, has driven the search for forage alternatives that are productive, resilient, and nutritionally valuable (Rivera et al., 2022; Ndung’u et al., 2023; Krüger et al., 2024).

*Tithonia diversifolia (Hemsl.) A. Gray*, commonly known as Mexican sunflower, is a shrubby species of high agroecological value, widely distributed in tropical regions. Several studies have highlighted its ability to thrive in poor and acidic soils, with high tolerance to low phosphorus levels and aluminum presence, making it suitable for fertility-limited conditions (Pazla et al., 2024). However, its aggressive growth and adaptability have also led to invasive behavior in some tropical regions, displacing native flora and altering ecosystems (Boral and Moktan, 2022).

Its high potential for biomass production in tropical silvopastoral systems has been demonstrated, due to its rapid growth rate, ease of propagation, and strong regrowth capacity (Rodríguez et al., 2017; Krüger et al., 2024; Angulo-Arizala et al., 2024). Moreover, T. *diversifolia* positively affects soil quality when used as green manure, accelerating mineralization and enhancing nutrient availability (Savón et al., 2017; Ruíz et al., 2024).

​ Nutritionally, this species offers a good crude protein content, acceptable digestibility (Castaño-Jiménez et al., 2023; Barreto-Cruz et al., 2024), and a complete profile of essential amino acids, including lysine, leucine, methionine, and valine (Olmo-González et al., 2022; Aye, 2016). Secondary metabolites such as tannins and total phenols remain at concentrations that do not compromise intake or digestibility (Gallego-Castro et al., 2017; Olmo-González et al., 2022; Pazla et al., 2024).

In animal feeding, trials with small ruminants fed *T. diversifolia* mixtures have shown improvements in feed conversion, digestibility, and carcass quality (Lezcano et al. (2024); Pazla et al., 2023; Huertas-González et al., 2023; Oyola-Zambrano, 2018). These findings support its inclusion in strategic diets for smallholders, particularly during the dry season (Del Pezo, 2022; Rivera-Herrera et al., 2021).

Despite its multiple advantages, most studies have focused only on the leaves, neglecting the stems and the integrated evaluation of total biomass for meal production (Rodríguez et al., 2017; Vargas-Velázquez et al., 2024).

Recent studies have reported that stems also contribute useful organic matter and do not compromise nutritive value when included in controlled proportions (Gallego-Castro et al., 2017; Ruíz et al., 2024).

Additionally, the effect of the planting system and harvest timing on plant chemical composition has been explored, with significant differences reported based on thermal time criteria (Angulo-Arizala et al., 2024). This climate-smart management approach has advantages in biomass yield and forage quality (Angulo-Arizala et al., 2024; Vargas-Velázquez et al., 2024).

There is consensus that harvest timing significantly affects leaf-to-stem ratio, crude protein concentration, fiber content, and energy value of the whole biomass (Rodríguez et al., 2017; Gallego-Castro et al., 2017; Theobald et al., 2014; Jamarun et al., 2024).

Therefore, determining the appropriate harvest stage and evaluating the total biomass of T. *diversifolia* is key to efficiently utilizing this plant for forage meal formulation (Jamarun et al., 2024), especially in tropical dry ecosystems where feed resources are limited (Arias-Gamboa et al., 2023; Vargas-Velázquez et al., 2024).

The aim of this study was to determine the optimal harvest stage of *Tithonia diversifolia* to maximize the yield and nutritive value of the meal derived from its total biomass under tropical dry forest conditions.

## 2 Materials and methods

### 2.1 Description of the study site

The study was carried out at the Los Cedros Experimental Station, part of the National Institute of Agricultural Innovation (INIA), located in the hamlet of Los Cedros, San Jacinto – Corrales district, Tumbes province and region, Peru. The experimental unit is geographically located at 3°37’41.85” S and 80°34’9.36” W, at an elevation of 6 meters above sea level.

Climatic characterization was based on data from the La Cruz Meteorological Station, the closest and most representative station for the study area. During the experimental period (June to August 2024), environmental conditions remained within the expected range for the dry season. The average monthly maximum temperature was 29.6 °C and the minimum was 23.7 °C, while precipitation was very low, ranging from 1.5 to 35 mm. Relative humidity averaged around 85%.

The soil presented a sandy loam texture (63% sand, 18% silt, and 19% clay), an alkaline pH of 7.5, and an electrical conductivity of 170 mS/m. Organic matter content was 1.02%, with available phosphorus at 8 ppm, potassium at 124 ppm, total nitrogen at 0.12%, and calcium carbonate (CaCO_3_) content at 0.52%.

### 2.2 Experimental design and sample processing

The study was conducted under a completely randomized design with a 3 × 3 factorial arrangement, considering harvest stage (30, 45, and 60 days of regrowth) and plant component type (leaves, stems, and a leaf-stem mixture) as the main factors. Each factorial combination was evaluated with four replicates, totaling 36 experimental units. This design was chosen based on the observed field homogeneity in soil texture, fertility, and topography, which minimized spatial variability. Under these conditions, the use of a Completely Randomized Design (CRD) was considered statistically appropriate for this experiment.

Experimental plots were established using *Tithonia diversifolia* stem cuttings derived from clonal banks maintained by INIA, using homogeneous vegetative material under uniform agronomic management conditions. The analyses were performed in duplicate and followed AOAC (2019) procedures, with internal quality assurance protocols that included the use of blanks, certified reference materials, and instrument calibration. Spacing was set at 1 meter between rows and 0.8 meters between plants, with an effective plot area of 18 m² per experimental unit. Each plot contained approximately 22–23 plants. Of these, ten plants were randomly selected for destructive sampling per treatment. This sampling intensity ensured that enough individuals remained to maintain plant density and spacing, avoiding interference in the growth of non-sampled plants. This plot size was selected based on prior forage research with *Tithonia* spp. and other tropical species under experimental station conditions. Although larger plots may offer broader extrapolation to farm-scale scenarios, the 18 m² area was adequate for assessing treatment effects under controlled and uniform agronomic conditions.

Biomass harvesting was performed at the three predetermined regrowth stages. Plant material was classified into leaves, stems, and a mixture of both components. Subsequently, biomass was dried at ambient temperature under shade with natural ventilation, on elevated mesh platforms, until reaching a constant weight. Drying duration varied depending on harvest stage, due to differences in moisture content. Once dried, the material was ground, homogenized, and 500-gram representative samples were obtained for each treatment (leaves, stems, and mixture), which were then sent to a certified private laboratory for bromatological analysis. Although temperature and humidity were not continuously monitored, the consistency of dry matter content across harvest stages (approximately 29%) indicates that the drying process was effective and did not compromise the integrity of the nutritional components.

### 2.3 Evaluated variables

Throughout the study, variables were grouped into three main categories: agronomic traits, productive performance, and nutritional quality. These categories were used to analyze the effect of harvest age on the morphophysiological development of *Tithonia diversifolia* and to evaluate its potential for producing high-quality forage meal.

First, agronomic variables related to vegetative growth were recorded through destructive sampling of ten randomly selected plants per experimental unit. The following variables were evaluated: plant height (cm), measured from the base to the apex of the main shoot; number of fully developed leaves per plant; stem diameter (mm), measured at the base using a digital caliper; and leaf area (cm²), estimated using ImageJ image analysis software from scanned leaf images obtained from representative subsamples.

Regarding productive performance, biomass yield was measured in different forms. Fresh forage yield (kg/m²) was recorded by weighing the total fresh biomass at harvest. The individual fresh weights of leaves and stems (kg/m²) were also recorded. Dry matter (DM) yield (kg/m²) was calculated after natural drying. Additionally, forage meal yield (g) from leaves, stems, and their mixture was determined based on 500 g of dry matter per treatment. Drying time (days) was also recorded for each sample, as this variable depended on the moisture content associated with harvest age.

Finally, to assess nutritional quality, samples of leaf, stem, and mixed forage meal were analyzed for the following bromatological parameters: dry matter (DM), crude protein (CP), crude fiber (CF), neutral detergent fiber (NDF), acid detergent fiber (ADF), ether extract (EE), total ash, and metabolizable energy (ME). Fiber fractions were determined using both the Weende method (for CF) and the Van Soest method (for NDF and ADF), with all procedures following AOAC (2019) standardized protocols. Analyses were performed in duplicate in a certified private laboratory, with routine quality control procedures including the use of blanks, standards, and calibrated equipment.

### 2.4 Statistical analysis

The data were subjected to analysis of variance (ANOVA) under a completely randomized design with a 3 × 3 factorial arrangement, corresponding to three harvest ages (30, 45, and 60 days) and three plant component types (leaves, stems, and mixture). The main effects of each factor and their interaction were evaluated on productive and nutritional variables. For agronomic traits, only harvest stage was considered as a factor, since plant component type was not applicable.

Prior to the analysis, the assumptions of normality and homogeneity of variances were verified using the Shapiro–Wilk and Levene tests, respectively. When required, data transformations were applied to meet model assumptions.

When statistically significant differences (p < 0.05) were detected in the ANOVA, means were separated using Tukey’s Honestly Significant Difference (HSD) test at a 5% significance level. This *post-hoc* method is appropriate for balanced factorial designs and controls the family-wise error rate. Therefore, additional corrections for multiple testing (e.g., Bonferroni or FDR) were not applied.

## 3 Results

### 3.1 Agronomic performance of *Tithonia diversifolia*

The morphological traits of *Tithonia diversifolia* exhibited significant variation according to harvest stage (30, 45, and 60 days after regrowth), demonstrating continuous and vigorous growth under arid–dry ecosystem conditions of northern Peru.

**Figure 1:**
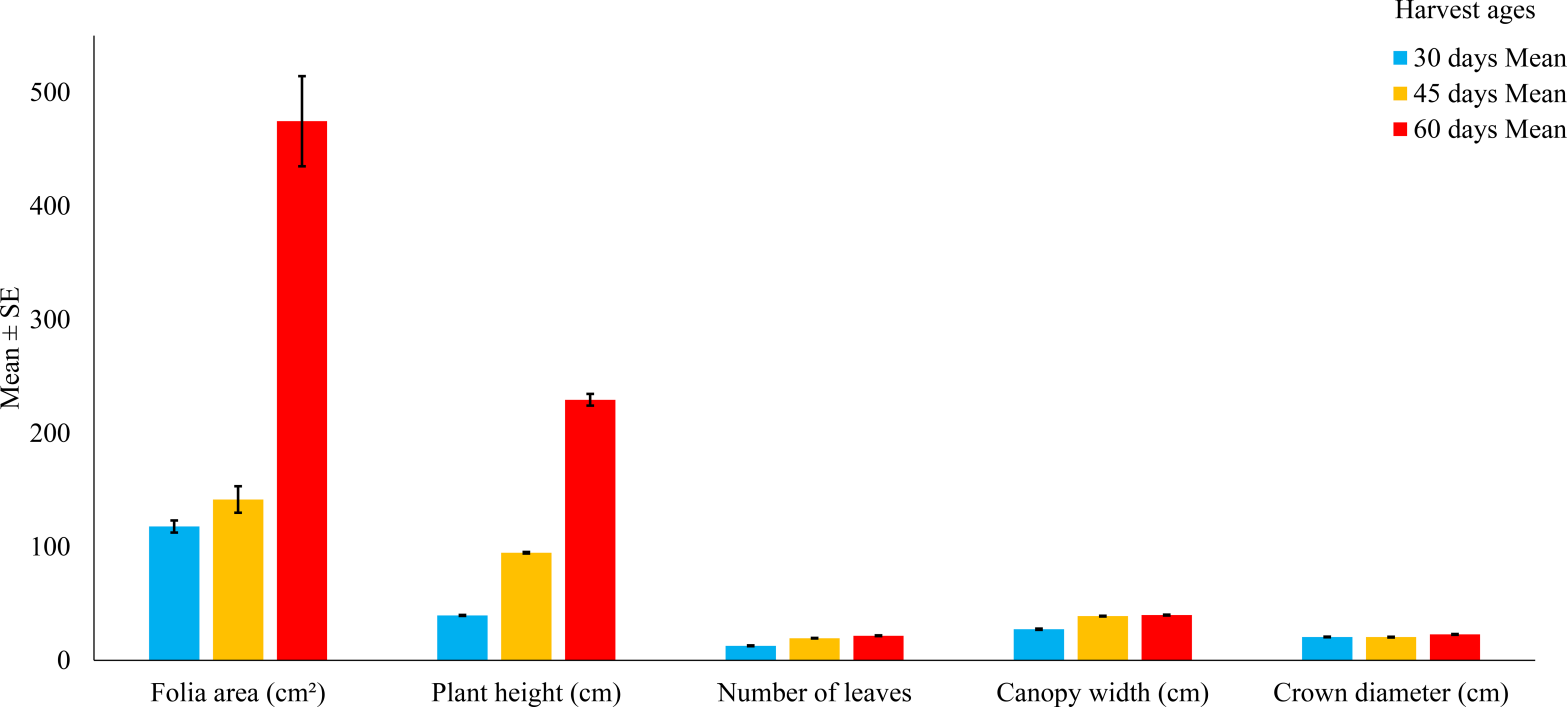
Agronomic variables of *Tithonia diversifolia* at different harvest ages (30, 45, and 60 days). Bars represent the mean ± standard error. According to ANOVA followed by Tukey’s test, significant differences were found between treatments (p < 0.001) for all variables.

Plant height increased from 39.90 ± 1.72 cm at 30 days to 97.11 ± 4.36 cm at 45 days, reaching 224.13 ± 13.35 cm at 60 days (**Figure 1**). This vertical growth was accompanied by a substantial increase in stem diameter, from 0.51 ± 0.07 mm (30 days) to 10.20 ± 0.18 mm (45 days) and 11.94 ± 0.54 mm (60 days), indicating structural reinforcement of the plant’s main axis.

The number of leaves per plant rose from 12.87 ± 0.60 at 30 days to 19.29 ± 0.49 (45 days) and 21.86 ± 0.69 at 60 days. This increase suggests greater photosynthetic capacity and potential for foliar biomass accumulation as regrowth progresses.

Leaf area also showed marked and progressive expansion: 114.05 ± 12.28 cm² at 30 days, 155.56 ± 24.01 cm² at 45 days, and 424.85 ± 114.31 cm² at 60 days, with highly significant differences between treatments (p < 0.001). This trend reflects rapid leaf development during early vegetative stages.

Lateral expansion was reflected in the increase in canopy width (26.69 ± 1.30 cm to 39.91 ± 1.35 cm) and crown diameter (20.39 ± 0.47 cm to 22.10 ± 1.18 cm), both showing significant variation across harvest stages.

All traits presented highly significant differences (p < 0.001), as confirmed by ANOVA and Tukey’s test (**Table 1**).

**Table 1:** Morpho-agronomic traits of *Tithonia diversifolia* evaluated at different harvest stages (30, 45, and 60 days) under arid–dry ecosystem conditions in northern Peru.

Variables	30 days	45 days	60 days	Statistical significance
Leaf area (cm²)	114.05 ± 12.28 c	155.56 ± 24.01 b	424.85 ± 114.31 a	***
Plant height (cm)	39.90 ± 1.72 c	97.11 ± 4.36 b	224.13 ± 13.35 a	***
Number of leaves	12.87 ± 0.60 c	19.29 ± 0.49 b	21.86 ± 0.69 a	***
Stem diameter (mm)	0.51 ± 0.07 c	10.20 ± 0.18 b	11.94 ± 0.54 a	***
Canopy width (cm)	26.69 ± 1.30 c	39.19 ± 0.89 b	39.91 ± 1.35 a	***
Crown diameter (cm)	20.39 ± 0.47 b	20.04 ± 0.96 c	22.10 ± 1.18 a	**

Values represent mean ± standard deviation. Different letters within the same row indicate statistically significant differences between harvest stages according to Tukey’s test (p < 0.05). ***p < 0.001; **p < 0.01.

### 3.2 Biomass yield

**Figure 2:**
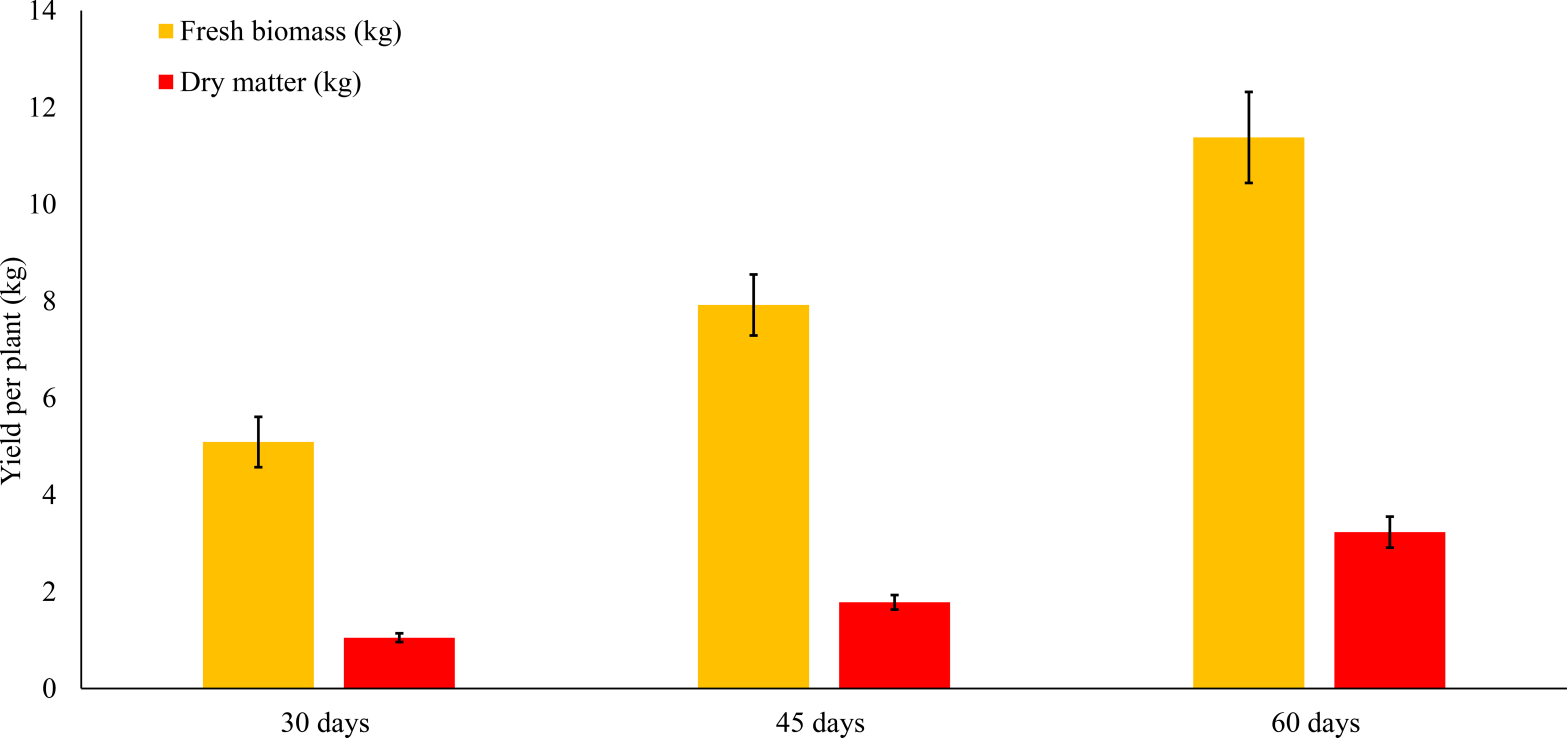
Fresh and dry matter yield of *Tithonia diversifolia* by harvest age.

Biomass yield of *Tithonia diversifolia* varied significantly with harvest age, showing an upward trend in both fresh biomass production and dry matter accumulation. These results are summarized in **Table 2** and illustrated in **Figure 2**.

**Table 2:** Statistical summary of biomass variables of *Tithonia diversifolia*.

Variable	30 days	45 days	60 days	Statistical significance
Total biomass (kg)	5.2 ± 1.31 c	7.88 ± 1.77 b	11.93 ± 2.3 a	***
Leaf weight (kg)	1.57 ± 0.31 c	2.31 ± 0.56 a	2.21 ± 0.33 b	**
Stem weight (kg)	3.64 ± 1.02 c	5.57 ± 1.3 b	9.71 ± 2.03 a	***
Total DM (kg)	1.51 ± 0.39 c	2.28 ± 0.48 b	3.45 ± 0.67 a	***
DM content (%)	28.9 ± 0.8 c	29.07 ± 0.98 a	28.95 ± 1.27 b	ns

Values represent mean ± standard deviation by harvest stage. Different letters in the same row indicate statistically significant differences according to Tukey’s test (p < 0.05). **p < 0.01; ***p < 0.001; ns, not significant.

Total fresh biomass per plant increased significantly as the regrowth period progressed, averaging 5.2 ± 1.31 kg at 30 days, 7.88 ± 1.77 kg at 45 days, and 11.93 ± 2.3 kg at 60 days (p < 0.001). This reflects the exponential growth pattern of the species during early phenological stages, where biomass accumulation is more efficient due to high photosynthetic activity and lower tissue lignification.

Similarly, dry matter (DM) yield increased significantly with cutting age, from 1.51 ± 0.39 kg (30 days) to 2.28 ± 0.48 kg (45 days) and 3.45 ± 0.67 kg (60 days) (p < 0.001). The highest absolute DM content recorded at 60 days suggests this stage is optimal for maximizing usable forage volume, although this must be complemented with nutritional quality data.

Structural component analysis revealed that stem weight increased significantly (p<0.001), from 3.64 ± 1.02 kg (30 days) to 9.71 ± 2.03 kg (60 days), indicating robust development of the plant’s main axis and support structure. In contrast, leaf weight did not follow a linear pattern: it peaked at 45 days (2.31 ± 0.56 kg) but slightly declined at 60 days (2.21 ± 0.33 kg), with statistically significant differences (p = 0.0073). This behavior may be attributed to leaf senescence or nutrient redistribution toward lignified or reproductive structures.

Regarding dry matter content (%DM), no significant differences were found between treatments (p = 0.9527), remaining relatively stable at around 29%. This suggests that, although total DM increases over time, its proportion relative to fresh biomass remains constant—likely due to a physiological balance between cellular water content and tissue structural composition.

### 3.3 Nutritional quality

The bromatological characterization of *Tithonia diversifolia*, considering the combined effect of harvest age (30, 45, and 60 days) and plant part (leaf, stem, and leaf–stem mixture), revealed highly significant differences (p < 0.001) in most of the variables evaluated, as shown in **Table 3**.

**Table 3:** Proximate and nutritional composition of Tithonia diversifolia by harvest age and plant part.

Treatment	EE	CP	ADF	NDF	IVDMD	DM	CA	CF	CHOS	OM	GE	ECHOS	EEE	EPC
30 days – Leaf	18.17 ± 13.72 d	16.54 ± 0.26 b	15.11 ± 0.31 h	8.87 ± 0.0 i	71.9 ± 8.63 a	18.3 ± 0.2 i	9.4 ± 0.1 c	19.3 ± 0.2 h	48.2 ± 0.3 c	90.6 ± 0.1 g	4.15 ± 0.05 h	2.45 ± 0.05 b	0.75 ± 0.05 a	0.65 ± 0.05 g
30 days – Leaf and Stem	11.73 ± 1.09 f	5.96 ± 0.06 e	38.72 ± 1.65 e	31.68 ± 0.86 f	59.96 ± 3.16 e	23.8 ± 0.3 f	7.2 ± 0.1 d	28.65 ± 0.35 e	46.0 ± 0.2 d	92.8 ± 0.1 f	4.35 ± 0.05 f	2.25 ± 0.05 d	0.65 ± 0.05 c	1.45 ± 0.05 d
30 days – Stem	5.48 ± 2.1 i	2.26 ± 0.1 i	54.18 ± 0.62 a	42.5 ± 0.04 c	43.68 ± 6.97 i	26.25 ± 0.45 c	6.0 ± 0.1 g	33.9 ± 0.2 a	42.8 ± 0.3 g	94.0 ± 0.1 c	4.55 ± 0.05 d	2.05 ± 0.05 g	0.55 ± 0.05 e	1.75 ± 0.05 a
45 days – Leaf	34.05 ± 0.56 a	16.58 ± 0.3 a	14.38 ± 0.72 i	9.8 ± 0.17 h	62.34 ± 1.42 c	19.6 ± 0.6 h	9.95 ± 0.15 b	19.05 ± 0.15 i	49.0 ± 0.3 a	90.05 ± 0.15 h	4.05 ± 0.05 i	2.65 ± 0.05 a	0.75 ± 0.05 b	0.55 ± 0.05 h
45 days – Leaf and Stem	13.2 ± 2.92 e	5.6 ± 0.6 f	40.9 ± 0.1 d	37.44 ± 0.41 d	61.96 ± 1.8 d	24.4 ± 0.5 e	6.6 ± 0.2 f	27.75 ± 0.25 f	45.05 ± 0.15 f	93.4 ± 0.2 d	4.55 ± 0.05 e	2.15 ± 0.05 f	0.55 ± 0.05 f	1.25 ± 0.05 f
45 days – Stem	10.88 ± 1.39 g	3.87 ± 0.7 h	46.75 ± 1.95 b	44.44 ± 6.2 a	55.06 ± 2.98 h	27.9 ± 0.4 b	5.5 ± 0.2 i	32.75 ± 0.25 c	41.25 ± 0.35 i	94.5 ± 0.2 a	4.75 ± 0.05 b	1.85 ± 0.05 i	0.45 ± 0.05 h	1.65 ± 0.05 c
60 days – Leaf	19.9 ± 7.32 c	10.6 ± 5.5 d	28.98 ± 13.52 f	22.28 ± 12.82 g	55.5 ± 4.59 g	20.7 ± 0.4 g	10.1 ± 0.2 a	19.8 ± 0.2 g	48.95 ± 0.15 b	89.9 ± 0.2 i	4.25 ± 0.05 g	2.45 ± 0.05 c	0.65 ± 0.05 d	0.55 ± 0.05 i
60 days – Leaf and Stem	20.42 ± 7.13 b	11.13 ± 5.06 c	26.3 ± 10.7 g	33.08 ± 2.84 e	56.72 ± 6.14 f	24.95 ± 0.25 d	7.05 ± 0.05 e	28.95 ± 0.25 d	45.75 ± 0.25 e	92.95 ± 0.05 e	4.75 ± 0.05 c	2.25 ± 0.05 e	0.55 ± 0.05 g	1.35 ± 0.05 e
60 days – Stem	7.4 ± 0.24 h	4.24 ± 1.12 g	44.3 ± 5.07 c	42.64 ± 6.4 b	63.23 ± 0.07 b	28.9 ± 0.2 a	5.9 ± 0.1 h	33.75 ± 0.25 b	42.35 ± 0.35 h	94.1 ± 0.1 b	4.95 ± 0.05 a	2.05 ± 0.05 h	0.45 ± 0.05 i	1.75 ± 0.05 b
p-value	NS	*	**	*	NS	***	***	***	***	***	**	***	**	***

Mean ± standard error values for ether extract (EE), crude protein (CP), neutral detergent fiber (NDF), acid detergent fiber (ADF), in vitro dry matter digestibility (IVDMD), as well as moisture, crude ash (CA), crude fiber (CF), soluble carbohydrates (CHOS), organic matter (OM), gross energy (GE), and estimated energy from carbohydrates (ECHOS), fat (EEE), and protein (EPC). Different superscript letters within the same row indicate significant differences according to Tukey’s test (p < 0.05). ***p < 0.001; **p < 0.01; *p < 0.05; ns, not significant.

Dry matter (DM) content increased with harvest age, especially in the stem fraction, which reached the highest values (up to 28.9 ± 0.2% at 60 days). Leaves, on the other hand, maintained lower and more stable DM values, with a minimum of 18.3 ± 0.4% at 30 days. Mixtures showed intermediate values, determined by the relative proportion of each component. This trend reflects progressive structural biomass development and reduced water content as physiological maturity advances.

Crude ash (CA), an indirect indicator of mineral content, was highest in young leaves (9.4 ± 0.1%) and decreased with advancing harvest age and transition to more lignified tissues such as stems. This consistent decline across treatments clearly distinguishes between metabolically active and structural tissues.

Crude fiber (CF) content differed markedly between leaves and stems. Stems presented the highest values (up to 33.75 ± 0.2%), regardless of age, with a slight increase at 60 days. Leaves maintained lower fiber levels, with a minimum around 19.3 ± 0.2% at 30 days. Mixtures showed intermediate values, generally closer to the foliar fraction at earlier stages.

**Figure 3:**
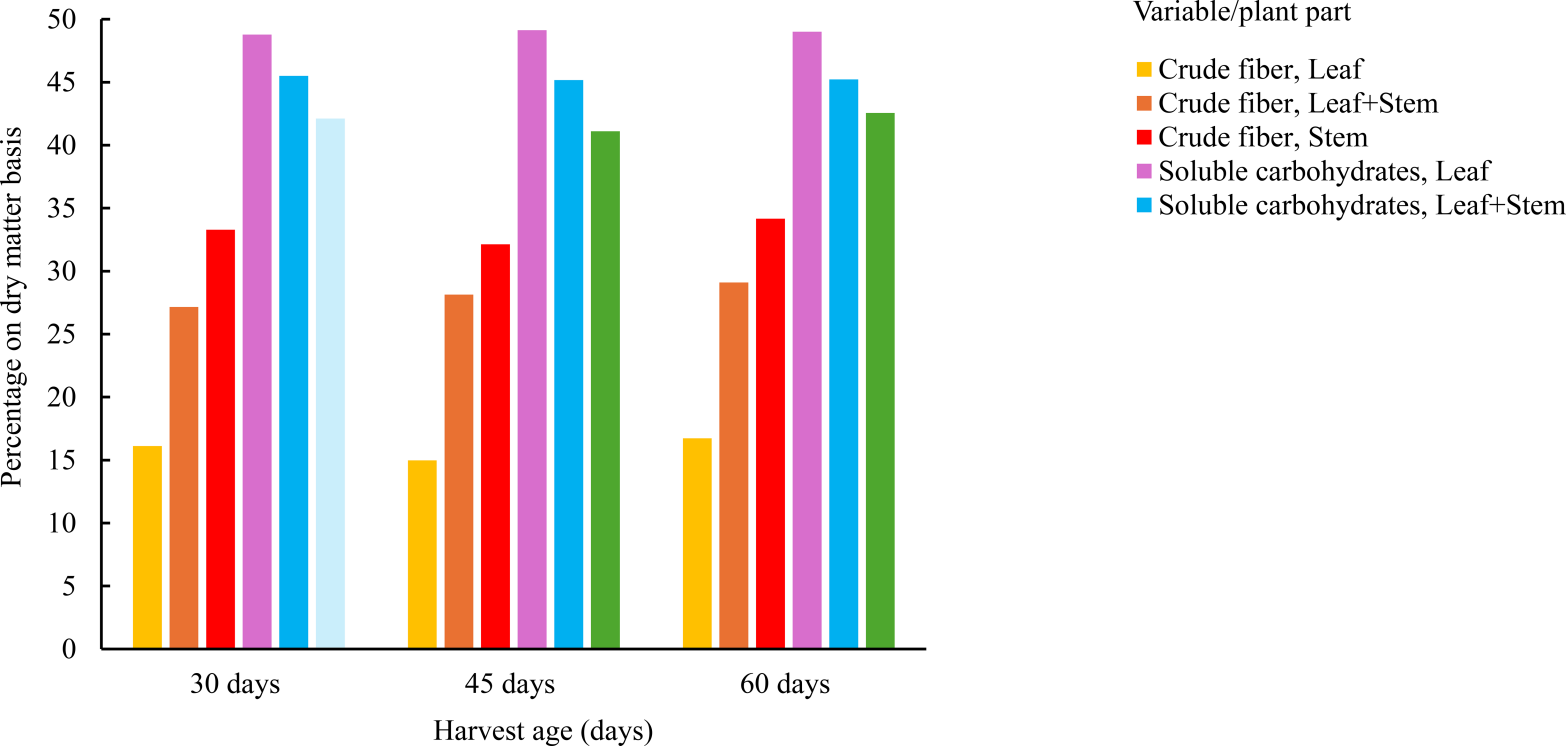
Average crude fiber and soluble carbohydrate content in *Tithonia diversifolia* by harvest age (30, 45, and 60 days) and plant part (Leaf, Stem, and Leaf + Stem).

Soluble carbohydrate (CHOS) content was noticeably higher in young leaves (48.2 ± 0.2%) and decreased significantly in stems (below 43%) and mixtures, particularly at 60 days. This trend was consistent across groups, showing a progressive decline with increasing physiological maturity. As shown in **Figure 3**, an inverse relationship was observed between CF and CHOS content based on harvest age and plant part. As the plant matures, the proportion of structural fiber increases, particularly in stems, while the soluble sugar fraction decreases markedly. Young leaves are characterized by low fiber and high available carbohydrate content, suggesting a highly digestible profile. Mixtures exhibited an intermediate pattern between both extremes.

Organic matter (OM) content remained high (>89%) in all treatments. However, slight but significant differences were observed depending on tissue type and harvest age, with the highest values found in 45-day stems (94.5 ± 0.1%) and the lowest in young leaves (90.6 ± 0.1%). This indicates minor variation in the proportion of combustible organic compounds as tissue physiology evolves.

A factorial two-way ANOVA was conducted to evaluate the effects of harvest stage (H), plant part (P), and their interaction (H × P) on nutritional traits. The full ANOVA results, including F-values, degrees of freedom, and p-values, are presented in **Supplementary Table S1**. Significant interaction effects were detected for most variables and are discussed in the main text.

Mean ± standard error values for ether extract (EE), crude protein (CP), neutral detergent fiber (NDF), acid detergent fiber (ADF), *in vitro* dry matter digestibility (IVDMD), as well as moisture, crude ash (CA), crude fiber (CF), soluble carbohydrates (CHOS), organic matter (OM), gross energy (GE), and estimated energy from carbohydrates (ECHOS), fat (EEE), and protein (EPC). Different superscript letters within the same row indicate significant differences according to Tukey’s test (p < 0.05).Regarding gross energy (GE), values ranged from 4.15 to 4.95 kcal/kg, being highest in mature stems (especially at 60 days). Leaves showed slightly lower but stable values, while mixtures followed the stem trend when that component was dominant.

Analysis of specific energy fractions showed that energy derived from carbohydrates (ECHOS) was highest in young leaves (2.45 ± 0.05 kcal/kg), with significant reductions in stems (down to 2.05 ± 0.05 kcal/kg). Fat-derived energy (EEE) was also higher in leaves (0.75 ± 0.05 kcal/kg), decreasing in stems with age. In contrast, energy from protein (EPC) increased in stems (up to 1.75 ± 0.05 kcal/kg at 60 days), consistent with a higher proportion of structural proteins and lower levels of soluble protein in mature leaves.

The chemical composition of *Tithonia diversifolia* varied significantly (p < 0.05) with both harvest age and plant component. Leaves consistently presented better nutritional indicators compared to stems, while mixtures showed intermediate values. A clear pattern of reduced protein content and increased structural fiber was observed with advancing physiological maturity.

### 3.4 Correlations between agronomic traits, yield, and nutritional quality

**Figure 4:**
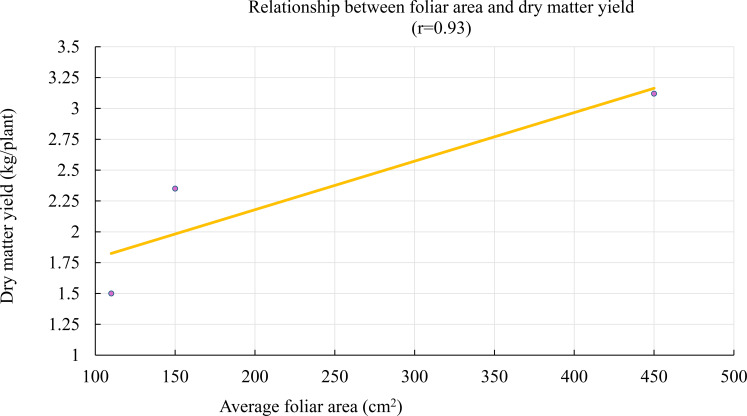
“Relationship between leaf area and dry matter yield of *Tithonia diversifolia*. A positive linear fit (r = 0.93) is observed, highlighting the contribution of leaf development to the increase in accumulated biomass.

A strong positive correlation was observed between average leaf area and dry matter (DM) yield (r = 0.93) (**Figure 4**). No influential outliers were detected in this correlation analysis, and the relationship remained robust after visual inspection and statistical validation. This result suggests that leaf expansion plays a key role in *Tithonia diversifolia*’s ability to accumulate biomass, by increasing the available photosynthetic surface area. Therefore, leaf area could serve as an early morphophysiological indicator of potential yield under extensive management conditions.

**Figure 5:**
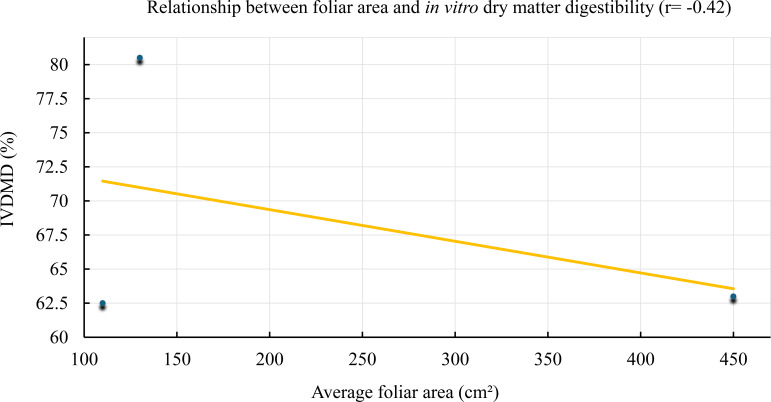
Relationship between average leaf area and *in vitro* dry matter digestibility (IVDMD) in *Tithonia diversifolia*. A decreasing trend is observed with increased structural foliar biomass (r = –0.42).

In contrast, a moderate negative correlation (r = –0.42) was observed between average leaf area and *in vitro* dry matter digestibility (IVDMD), as shown in **Figure 5**. This trend indicates that as average leaf area increases, digestibility tends to decline.

Although the correlation was not strong, it suggests that treatments with larger leaf area—associated with more advanced regrowth stages—exhibited lower ruminal degradability, possibly due to higher structural fiber content or progressive tissue lignification. On the other hand, treatments with smaller leaf area (typically younger leaves) showed higher IVDMD values, exceeding 80%, highlighting their high nutritional value and potential as a strategic component in highly digestible diets.

## 4 Discussion

The results obtained in this study confirm that harvest age has a decisive effect on the morphological, productive, and nutritional variables of *Tithonia diversifolia*, a species of growing interest for its forage potential in arid-zone production systems. In addition to its agronomic value, this species is also recognized for its invasive capacity in non-native regions due to its dual reproductive strategy, rapid establishment, and aggressive growth under limited resource conditions (Muoghalu and Chuba, 2005). This influence is clearly reflected in the transformation of its vegetative structure, biomass accumulation, and the dynamics of its chemical components—dimensions that have been partially addressed in previous studies but are integrated and analyzed here comprehensively.

From a morpho-agronomic perspective, the increases in plant height, number of leaves, leaf area, and stem diameter with advancing regrowth age are consistent with the findings of Ovani et al. (2023); Sabaris et al. (2023); Krüger et al. (2024), and Angulo-Arizala et al. (2024), who attribute this trend to increased meristematic activity and cell elongation and adaptive responses even under suboptimal water availability. Our study provides concrete data under moderate water stress conditions, where the species still manages to develop robust structures, demonstrating its adaptability and photosynthetic efficiency, as previously reported by Theobald et al. (2014); Padilla Corrales and Rodríguez García (2021) and Li et al. (2024), who highlighted *T. diversifolia*’s rapid photosynthetic response and superior carbon gain under fluctuating light conditions. Recent multi-seasonal trials in Brazil have also confirmed the high biomass accumulation and favorable nutritional profile of T. *diversifolia* accessions across tropical biomes (Soares et al., 2024).

In terms of yield, a progressive increase was observed in both fresh and dry biomass, peaking at 60 days. This trend is well-documented in the literature (Rodríguez et al., 2017; Pazla et al., 2023), reflecting the typical pattern of fast-growing perennial species. However, our results show that this increase does not always translate into improved forage quality. The proportion of leaves decreased markedly in favor of more lignified stems, which is consistent with the findings of Angulo-Arizala et al. (2024), who noted a shift in biomass allocation towards structural tissues.

The reduction in crude protein (CP) content with age, particularly in the stem and mixed fractions, aligns with the findings of Nouel-Borges and Romero (2023), Takahashi et al. (2023), and Lezcano et al. (2024), who explain that nitrogen tends to be redirected to reproductive organs or storage tissues as the plant matures. Furthermore, our results show that while leaves maintain acceptable protein levels (>18%) even at 45 days, stem values may fall below 10%, limiting their usefulness in unsupplemented diets (Castaño-Jiménez et al., 2023; Jamarun et al., 2024).

Although the number of replicates (n = 4) was sufficient to detect statistically significant differences in most variables, we acknowledge that a higher replication level might improve the detection of subtle biological effects. The sample size was chosen based on logistical feasibility under field conditions and aligns with similar forage evaluation studies (Rodríguez et al., 2017).

Regarding fiber content, increases in NDF and ADF in stems exceeded 60%, values consistent with those reported by Rivera et al. (2022), Arias-Gamboa et al. (2023) for similar tropical forages. This explains the sharp drop in *in vitro* digestibility (IVDMD), which in our data decreased by more than 15 percentage points between 30 and 60 days. This is particularly relevant, reinforcing the importance of precise harvest timing when using the whole plant (Vargas-Velázquez et al., 2024; Castaño-Jiménez et al., 2023). Similar results were found by Hernández-Arboleda et al. (2024), who reported that differences in dry matter and fiber content among *Tithonia diversifolia* accessions affected *in vitro* gas production and fermentation characteristics, emphasizing the importance of genetic variability and harvest stage in optimizing forage quality.

The negative correlation between leaf area and digestibility (r = –0.42), although moderate, suggests that more developed leaves contain a greater proportion of structural tissues (e.g., midribs, veins), which could account for the loss in ruminal degradability, even within the leaf fraction (Vargas-Velázquez et al., 2024). This pattern has also been observed by Gallego-Castro et al. (2017) and Barreto-Cruz et al. (2024), who associated greater leaf biomass with lower fermentability due to increased cell wall components. This relationship has been scarcely addressed in previous studies and therefore represents a novel contribution of the present research.

Although the correlation analysis was based on a single season and a limited number of replicates, it was included as an exploratory approach to identify potential relationships between morphological and nutritional traits. These correlations, while moderate in some cases, offer useful biological insights and should be interpreted cautiously as hypothesis-generating trends under dry-season conditions. This contextualization has been reflected in the manuscript to clarify its scope.

We also found that the dry matter content (%DM) remained stable around 29% across treatments, suggesting that natural drying is not impaired by harvest age. This contrasts with the findings of López et al. (2022), who reported higher moisture retention in mature Moringa oleifera plants, thus highlighting a comparative advantage of *T. diversifolia* in terms of post-harvest management.

In terms of energy, our data show a decrease in gross energy (GE) and energy estimated from soluble carbohydrates (E-SCHO) with plant maturity, matching the reduction in soluble carbohydrate concentrations. This trend is consistent with the findings of Gallego-Castro et al. (2017), Nur Adli et al. (2023), and Pazla et al. (2024), and underscores that younger tissues are more metabolically active and energy-dense—reinforcing their value in diets for animals in physiologically demanding stages such as growth or lactation.

From a practical perspective, harvesting between 45 and 60 days represents a viable balance between yield and quality, particularly when leaf-to-stem ratios are managed strategically. In goat production systems, where feeding efficiency is essential, the use of whole-plant *Tithonia* meal may be feasible as long as inclusion levels are properly adjusted or combined with energy-rich or high-fiber feed sources, as suggested by Rodríguez et al. (2017), Angulo-Arizala et al. (2024), Mauricio et al. (2017), and Pazla et al. (2023).

Recent findings by Souza et al. (2025) also reinforce the importance of determining the optimal harvest age for maximizing both yield and nutritional quality in T. *diversifolia*. Their results confirm that delayed harvest improves total biomass but compromises protein concentration and digestibility, consistent with the trade-offs observed in our study. These findings support our recommendation to harvest between 45 and 60 days as a balance between forage quantity and quality for ruminant diets.

A particularly relevant finding in this study is that *Tithonia diversifolia* meal—especially from mixtures with a predominance of leaf tissue—could be an excellent ingredient for the formulation of multinutritional blocks (Oyola-Zambrano, 2018). Its crude protein content, soluble carbohydrates, and dry matter profile make it a viable substitute, partially or fully, for conventional inputs such as soybean meal or legume-based feedstuffs, whose availability and price can limit access for small-scale producers. These nutritional advantages are further supported by findings from silage-based studies, where *T. diversifolia* inclusion improved protein levels and reduced methane emissions (Takahashi et al., 2023). Recent studies by Ovani et al. (2023), and Nouel-Borges and Romero (2023) support the use of non-conventional forages in supplementation blocks, emphasizing their positive impact on voluntary intake and animal performance under extensive grazing conditions.

Finally, from an ecological and sustainability standpoint, T. *diversifolia* stands out as a strategic resource. Its integration into silvopastoral systems not only improves forage supply but also contributes to soil conservation, carbon sequestration, and reduced enteric methane emissions—as demonstrated by Nuwarapaksha et al. (2024) and Ruíz et al. (2024). Its inclusion as a meal in multinutritional blocks would further enhance its value in sustainable livestock systems, integrating forage production, animal nutrition, and climate change adaptation. Moreover, its inclusion in silage mixtures has been shown to reduce *in vitro* methane production and improve protein availability, further reinforcing its environmental benefits in ruminant feeding systems (Takahashi et al., 2023).

## 5 Conclusion

This study provides new integrative evidence on how harvest age and plant fraction affect the morphological development, biomass accumulation, and nutritional profile of *Tithonia diversifolia*. Beyond confirming general trends in forage maturation, our results demonstrate that despite a reduction in crude protein and digestibility, the dry matter content remains stable across ages, supporting its suitability for natural drying. We identified 45 to 60 days as an optimal harvest window to balance yield and quality, especially when whole-plant meal is used strategically. Additionally, the potential of *T. diversifolia* as an ingredient for multinutritional blocks is reinforced by its high protein content in leaf-dominant mixtures and favorable post-harvest characteristics. These findings contribute to more informed and sustainable decisions regarding the use of this forage resource in arid tropical livestock systems.

## Data Availability

The original contributions presented in the study are included in the article/**Supplementary Material**. Further inquiries can be directed to the corresponding author.
