# Supplementary material for: Optimizing harvest stage and drying time to enhance yield and nutritive quality of whole-plant Tithonia diversifolia forage meal in arid tropics
Source: Front Plant Sci. 2025 Sep 19;16:1644949. doi: 10.3389/fpls.2025.1644949 (PMC12491292; doi:10.3389/fpls.2025.1644949)
Supplement: Supplementary file 1 [file DataSheet1.pdf]

Supplementary Table S1. Two-way ANOVA summary results for the effects of harvest stage, plant part, and their interaction on nutritional traits of Tithonia diversifolia.

**Variable**	**Source**	**Sum of Squares**	**Degrees of Freedom**	**F-value**	**p-value**
PC	Harvest Stage (H)	5.03	3	0.24	0.792
PC	Plant Part (P)	529.50	2	37.99	0.000
PC	H * P	220.68	6	5.28	0.022
PC	Residual	55.76	8		
FC	Harvest Stage (H)	33.92	3	0.31	0.744
FC	Plant Part (P)	3269.58	2	44.27	0.000
FC	H * P	1431.33	6	6.46	0.013
FC	Residual	295.39	8		
FDA	Harvest Stage (H)	3.83	3	0.03	0.968
FDA	Plant Part (P)	1992.28	2	25.39	0.001
FDA	H * P	1075.12	6	4.57	0.033
FDA	Residual	313.86	8		
FDN	Harvest Stage (H)	35.50	3	0.38	0.696
FDN	Plant Part (P)	2591.30	2	41.50	0.000
FDN	H * P	1479.76	6	7.90	0.007
FDN	Residual	249.76	8		
CHOS	Harvest Stage (H)	277.26	3	1.38	0.306
CHOS	Plant Part (P)	317.18	2	2.37	0.163
CHOS	H * P	162.30	6	0.40	0.801
CHOS	Residual	536.32	8		
DIVMS	Harvest Stage (H)	27.97	3	0.08	0.925
DIVMS	Plant Part (P)	962.76	2	4.06	0.079
DIVMS	H * P	517.55	6	0.73	0.598
DIVMS	Residual	949.39	8		

Supplementary Table S1. Results of the two-way ANOVA assessing the main effects of harvest stage (H), plant part (P), and their interaction (H × P) on nutritional traits of Tithonia diversifolia. The table presents the sum of squares, degrees of freedom, F-values, and p-values for each source of variation. Significant p-values ($p < 0.05$) are highlighted in bold in the manuscript. Data correspond to four replicates per treatment combination.
